# Supplementary material for: Transfer learning with graph neural networks for improved molecular property prediction in the multi-fidelity setting
Source: Nat Commun. 2024 Feb 26;15:1517. doi: 10.1038/s41467-024-45566-8 (PMC11258334; doi:10.1038/s41467-024-45566-8)
Supplement: Supplementary file 1 — Supplementary Information [file 41467_2024_45566_MOESM1_ESM.pdf]

Transfer learning with graph neural networks for improved molecular property prediction in the multi-fidelity setting
Supplementary Information

David Buterez*[,1], Jon Paul Janet[2], Steven J. Kiddle[3], Dino Oglic[4], and Pietro Liò[1]

[1]Department of Computer Science and Technology, University of Cambridge, Cambridge, UK
*Corresponding author (`db804@cam.ac.uk`)
[2]Molecular AI, BioPharmaceuticals R&D, AstraZeneca, Gothenburg, Sweden
[3]Data Science & Advanced Analytics, Data Science & AI, R&D, AstraZeneca, Cambridge, UK
[4]Centre for AI, BioPharmaceuticals R&D, AstraZeneca, Cambridge, UK

Supplementary Notes 1 Summary of PubChem datasets

Supplementary Table 1. Summary of the 23 public multi-fidelity high-throughput screening (HTS datasets), including the PubChem AID, SD and DR measurement types, assay type, size of the datasets (denoted by #), the Pearson correlation coefficient (r) for the paired SD/DR measurements, and the associated *p* value. If the confirmatory data is available separately, both AID columns are populated, otherwise the SD dataset includes the DR data. The first 8 rows represent our starting set of public multi-fidelity data, the following 10 rows correspond to the datasets with the highest SD/DR correlation, and the last 5 rows summarise datasets that were added to support our analysis for compounds that have DR measurements but lack primary screening data. The shortened words stand for: Inh., Inhibition; Act., Activation; Ind., Induction, FP, fluorescence polarisation. The abbreviations are: AID, assay identifier; SD, single dose; DR, dose response. Bold is used for the table headers.

**DR AID**	**SD AID**	**SD type**	**DR type**	**Assay type**	**# SD**	**# DR**	**r**	**p value**
1259350	1224905	Z-score	FP	Protein	202,486	569	0.41	2.11×10^{-24}
1259418	1259416	Act.	pAC50	Organism	59,447	711	−0.37	1.97×10^{-24}
449756	435005	% change in signal	LogAC50	Cell	289,447	1,811	0.25	3.59×10^{-27}
–	449762	Inh. @25 μM	IC50	Cell	311,910	1,754	0.20	6.04×10^{-18}
–	1465	Fold Ind. @50 μM	EC50	Cell	205,193	980	−0.14	1.72×10^{-5}
1259375	1259374	Inh. @2.6 μM	LogIC50	Protein	614,427	348	0.10	6.89×10^{-2}
–	1949	Inh. @10 μg/mL	IC50 (μg/mL)	Cell	98,472	1,688	0.09	9.48×10^{-05}
1431	873	Inh. @5 μM	IC50	Protein	204,361	1,215	0.08	8.22×10^{-3}
–	504329	Inh. @12.5 μM	IC50	Protein	319,080	902	0.79	7.85×10^{-192}
–	1445	Inh. @30 μM	IC50	Protein	207,096	655	0.78	6.06×10^{-137}
624273	588549	Act. @12.48 μM	pAC50	Protein	337,483	359	0.70	1.55×10^{-54}
624326	602261	Act. @15 μM	IC50	Protein	343,811	985	0.68	1.03×10^{-133}
–	624330	Inh. @30 μM	IC50	Protein	324,979	1,570	0.66	2.30×10^{-198}
504941	488895	Act.	pAC50	Cell	321,242	161	0.63	4.15×10^{-19}
720512	652162	Act. @9.99 μM	pAC50	Protein	264,972	109	0.62	9.55×10^{-13}
624474	624304	Inh. @21.8 μM	IC50	Organism	345,553	1,327	0.58	1.43×10^{-121}
493155	485273	Inh. @20 μM	IC50	Protein	314,791	973	0.58	3.80×10^{-88}
435010	2221	Act.	LogEC50	Protein	280,006	1,797	0.56	1.27×10^{-149}
463203	2650	Act. @10 μM	LogAC50	Protein	300,560	721	0.42	1.83×10^{-31}
1259420	1259416	Act.	pAC50	Organism	59,447	174	−0.28	1.86×10^{-4}
2382	2098	Act. @7.5 μM	EC50	Cell	287,633	2,239	−0.24	1.29×10^{-29}
687027	652154	Act. @12.62 μM	pAC50	Cell	281,074	1,024	0.10	1.72×10^{-3}
504313	2732	Inh. @10 μM	IC50	Cell	208,123	855	−0.09	5.84×10^{-3}

Supplementary Notes 2 Summary of AstraZeneca datasets

Supplementary Table 2. Summary of the AstraZeneca multi-fidelity high-throughput screening (HTS) datasets, including the SD and DR dataset names, SD and DR measurement types, size of the datasets (denoted by #), the Pearson correlation coefficient (r) for regression datasets or the point-biserial correlation coefficient (classification datasets) for the paired SD/DR measurements, and the associated *p* value. It is possible for the same primary screening (SD) dataset to be associated with multiple confirmatory (DR) datasets. A value of '$\leq \epsilon$' denotes an extremely low value that is below the used machine precision. Act., Activation. The abbreviations are: AZ, AstraZeneca, R, regression; C, classification; SD, single dose; DR, dose response. Bold is used for the table headers.

**DR name**	**SD name**	**SD type**	**DR type**	**# SD**	**# DR**	**r**	**p value**
AZ-DR-R1	AZ-SD1	Z-Score	pIC50	1,700,745	6,522	−0.77	$\leq \epsilon$
AZ-DR-R2	AZ-SD2			1,676,309	3,420	−0.72	$\leq \epsilon$
AZ-DR-R3	AZ-SD3			1,970,086	9,654	−0.67	$\leq \epsilon$
AZ-DR-R4 R2	AZ-SD4			1,370,897	914	−0.66	9.71×10^{-110}
AZ-DR-R5	AZ-SD3			1,970,086	9,523	−0.66	$\leq \epsilon$
AZ-DR-R6	AZ-SD5			1,360,029	3,467	−0.66	$\leq \epsilon$
AZ-DR-R2	AZ-SD6			1,013,581	11,828	−0.64	$\leq \epsilon$
AZ-DR-R4 R1	AZ-SD4			1,370,897	1,073	−0.58	1.14×10^{-68}
AZ-DR-R7	AZ-SD7			1,742,284	7,416	−0.53	$\leq \epsilon$
AZ-DR-R8	AZ-SD7			1,742,284	6,909	−0.49	$\leq \epsilon$
AZ-DR-R9	AZ-SD8			1,753,721	10,091	−0.46	$\leq \epsilon$
AZ-DR-R10	AZ-SD9			1,581,928	399	−0.33	8.61×10^{-11}
AZ-DR-R11	AZ-SD10			1,671,471	4,488	−0.30	1.05×10^{-83}
AZ-DR-R12	AZ-SD11			1,747,502	5,642	−0.28	1.59×10^{-99}
AZ-DR-R13	AZ-SD11			1,747,502	4,698	−0.19	3.64×10^{-38}
AZ-DR-R14	AZ-SD12			1,962,638	6,511	−0.14	7.28×10^{-18}
AZ-SD13	AZ-DR-C1	Z-Score	Binary Act.	1,482,258	4,901	−0.22	3.50×10^{-52}
AZ-SD14	AZ-DR-C2			1,701,084	4,260	0.32	7.93×10^{-104}

Supplementary Notes 3 Summary of QMugs properties (10K diverse set)

Supplementary Table 3. Correlation (Pearson's r) between the low-fidelity GFN2-xTB and the high-fidelity DFT measurements for the QMugs dataset. The abbreviations are: DFT, density functional theory; GFN2-xTB, geometry, frequency, noncovalent, eXtended tight binding; HOMO, highest occupied molecular orbital; LUMO, lowest unoccupied molecular orbital. The shortened term 'Rot.' stands for 'Rotation'. Bold is used for the table headers.

**Property**	**DFT/GFN2-xTB r**
Atomic Energy	0.58
Dipole Total	0.97
Dipole X	0.99
Dipole Y	0.98
Dipole Z	0.98
Formation Energy	1.00
HOMO Energy	0.79
LUMO Energy	0.95
Rot. Constant A	1.00
Rot. Constant B	1.00
Rot. Constant C	1.00
Total Energy	0.56

Supplementary Notes 4 Train R^2 for low-fidelity models

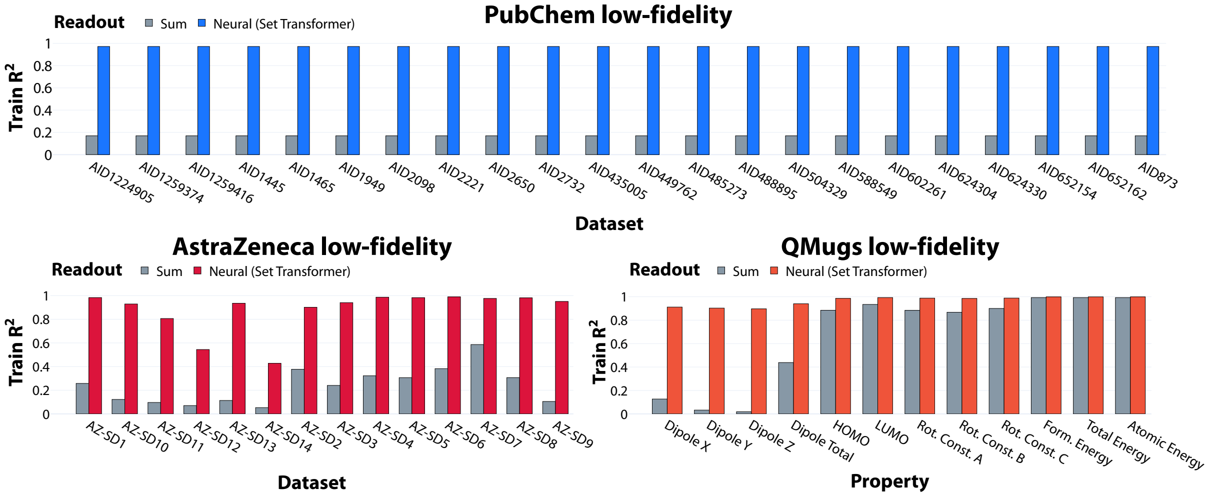

Supplementary Figure 1. Train R^2 for the PubChem, AstraZeneca, and QMugs low-fidelity models of Figures 3A and 3B. Source data are provided as a Source Data file.

Supplementary Notes 5 Data selection and filtering steps

The main challenge in the data curation comes from the fact that the SD and DR measurements are often reported as separate, independent datasets. In this work, when referring to a multi-fidelity dataset, the identifier of the DR dataset is written first, followed by the SD identifier and separated by '–', e.g. AID2382 – 2098 for public data (AID, assay identifier), or AID1445 if the SD and DR values are reported in the same assay. In this paper, each AstraZeneca dataset is assigned an arbitrary identifier such as AZ-SD1 for single dose, AZ-DR-R1 for DR (regression), and AZ-DR-C1 for DR (classification). These naming schemes are exemplified in Supplementary Tables 1 and 2.

PubChem datasets provide a CID (Compound ID) for each compound. However, multiple substances can have the same CID and to account for this we retrieve a SMILES (Simplified molecular-input line-entry system) string for each CID using the PubChem REST API. We use the open-source Python library RDKit to produce a `mol` object from the SMILES [1] with the `MolFromSmiles` function, which performs a sanitisation procedure internally and acts as the first filtering step. Multiple molecules associated with a CID are usually counter ions or salts, and these are dropped from the dataset. Subsequent filtering steps include the removal of stereoisomers (molecules with the same chemical formula but different spatial organisation) and charged species. Furthermore, molecules with more than 125 atoms are dropped. The same filtering steps are applied to the in-house AstraZeneca datasets. The pre-processing steps are presented in detail in [2].

Supplementary Notes 5.1 Diverse compounds selection

To select a diverse collection of molecules, we first represent them using the Morgan fingerprint obtained via the RDKit procedure that was also used to generate feature representations for classical methods such as random forests and support vector machines. We configure the Morgan fingerprint `GetMorganFingerprintAsBitVect()` function from RDKit with `radius=3` and `nBits=2048`. To select diverse compounds, we then use the `MaxMinPicker` class that is available from `rdSimDivPickers` via `SimDivFilters`. In particular, we use the `lazyPick()` function with a pairwise distance function based on the Dice similarity `d(m1, m2) = 1 - DataStructs.DiceSimilarity(m1, m2)`, where `m1`, `m2` are the fingerprint representations of two molecules. The remaining inputs to `lazyPick()` are the size of the dataset, the number of diverse molecules to select (e.g. 10,000), and a seed that was set to 0. We used these steps to select a diverse set of 10,000 molecules from the QMugs dataset. After selection, we used a `FingerprintSplitter` from the `deepchem` library to ensure a difficult train, validation, and test split of the data using a ratio of 80%/10%/10%.

Supplementary Notes 5.2 Hyperparameter selection

Support Vector Machines and Random Forests

For classical methods (i.e. support vector machines and random forests), we leverage the `sklearn` library and the methods: i) `svm.SVR` and `ensemble.RandomForestRegressor` in the case of regression, and ii) `svm.SVC` and `ensemble.RandomForestClassifier` in the case of classification. The inputs to these learning algorithms are Morgan fingerprints computed as described above for the selection of diverse compounds (Supplementary Notes 5.1).

The hyperparameters were optimised using grid search (i.e. `GridSearchCV` from `scikit-learn`) with negative mean squared error scoring for regression and the area under the receiver operating characteristic curve scoring for classification. For the hyperparameter search, the train, validation, and test folds were defined using `PredefinedSplit` from the `sklearn` library. For random forest baselines, the hyperparameter space is defined by `n_estimators` $\in \{50, 100, 150, 200, 250, 300, 500\}$, `max_depth` $\in$ {None, 25, 50, 100}, `min_samples_leaf` $\in \{1, 2, 4\}$ and `min_samples_split` $\in \{2, 5, 10\}$, whereas for the SVM models (SVR/SVC for regression/classification) the space is defined by `C` $\in$ `logspace(-2, 3, 10)` and `gamma` $\in$ `logspace(-5, 2, 20)`, using the open-source Python library `numpy`. The hyperparameter search was repeated for a variation where standard scaling (given by `preprocessing.StandardScaler()` in `scikit-learn`) was applied to the ground truth high-fidelity dose response scores (the scaler is trained only on the train set), and then the transformation was reversed when computing the metrics. Models using default hyperparameters for random forests and support vector machines where also considered (one for the unscaled outputs and one for standard scaling). In total, 4 configurations (two using default parameters and two using hyperparameter search, with and without scaling) were considered for each of the 2 input types (fingerprints, PhysChem descriptors), resulting in 8 configurations per algorithm (random forests or support vector machines). The total number of configurations per dataset is thus 16. For the reported results, the best out of the 8 configurations for each dataset and algorithm was selected according to the validation set performance. The full implementation is available in our code repository [3].

Graph Neural Networks

Deep learning models make use of PyTorch [4], PyTorch Geometric [5], and PyTorch Lightning [6], and thus follow the conventions used by these libraries. In addition to the graph structure of the molecules, the node features are calculated using the same approach as ChemProp [7–9]. The procedure concatenates one-hot encodings of: the atomic number, the number of directly-bonded neighbours, the formal charge, the chiral tag, the total number of bonded hydrogen atoms (implicit and explicit), and the hybridisation type. An aromatic binary flag and the (scaled) atom mass are then appended to this representation.

The final input size d_{in} depends on the largest atomic number encountered in each dataset, so the chosen approach is to use a large constant value. Here, we do not make use of edge features. As the deep learning models include a wider range of hyperparameters and require more resources to train, we do not perform traditional hyperparameter tuning for the supervised VGAEs that are discussed and presented in this paper. The full implementation is available in our code repository [3].

The default choice is to use 3 graph convolutional layers (GCN [10]) preceding the graph convolutional layers for each Gaussian parameter ($\boldsymbol{\mu}$, $\boldsymbol{\sigma}$) of the VGAE. For comparison purposes we fix a single set of hyperparameters for the low-fidelity models, following the architecture: $\text{GCN}_1 : (d_{\text{in}}, d_{\text{interim}})$, $\text{GCN}_2 : (d_{\text{interim}}, d_{\text{interim}})$, $\text{GCN}_3 : (d_{\text{interim}}, d_{\text{interim}})$, $\text{GCN}_\mu : (d_{\text{interim}}, d_{\text{out}})$, and $\text{GCN}_\sigma : (d_{\text{interim}}, d_{\text{out}})$. For low-fidelity models, we use a single readout function at a time: sum as a representative of standard readouts, and the attention-based Set Transformer as a neural (adaptive) readout. For high-fidelity models that generally operate on datasets with less than 10,000 molecules, we only use standard readouts (more specifically, sum) as we do not expect large improvements due to the data-driven nature of the Set Transformer. This choice also simplifies the analysis. To maximise the chance of learning high-quality representations from the sparse high-fidelity data, we pass the node representations through three standard readout functions separately (sum, mean, and maximum), and concatenate the outputs. This concatenated vector is used as input to the regression/classification multi-layer perceptron at the end of the model, potentially further concatenated with other data representations when using augmentations.

Otherwise, we make a series of hyperparameter choices based on empirical observations of the best performing models: a node latent dimension of 50, a learning rate of 0.00005, a batch size of 512 for the low-fidelity models and of 32 for the high-fidelity models, and a GCN intermediate dimension of 256. The low-fidelity models are always trained for a set number of epochs: 200 for the PubChem and QMugs datasets and 300 for the AZ datasets. High-fidelity models do not have a prespecified number of epochs and instead use an early stopping mechanism based on the validation set and with a patience of 100 epochs. The low-fidelity models using the sum readout function and the high-fidelity models have the same graph-level latent dimension as the node dimension, since the standard pooling operations do not change the dimensionality. In the case of the Set Transformer readout, however, the two dimensions are disentangled and we use a graph latent dimension of 256. Furthermore, the Set Transformer introduces a number of hyperparameters. For all experiments, we used a hidden dimension of 64, with 16 attention heads and 2 self-attention blocks (MABs with identical inputs) for the attention mechanism.

We have empirically not noticed improved performance by adding convolutional layers beyond our choice of 3, using edge (bond) features, or changing to more expressive graph layer types (e.g. GIN [11] or PNA [12]). These choices also significantly increase the computational burden and training time, making analyses on the scale presented here much more difficult.

Supplementary Notes 6 Systematic evaluation of transductive low-fidelity augmentations for drug discovery – AZ GNNs

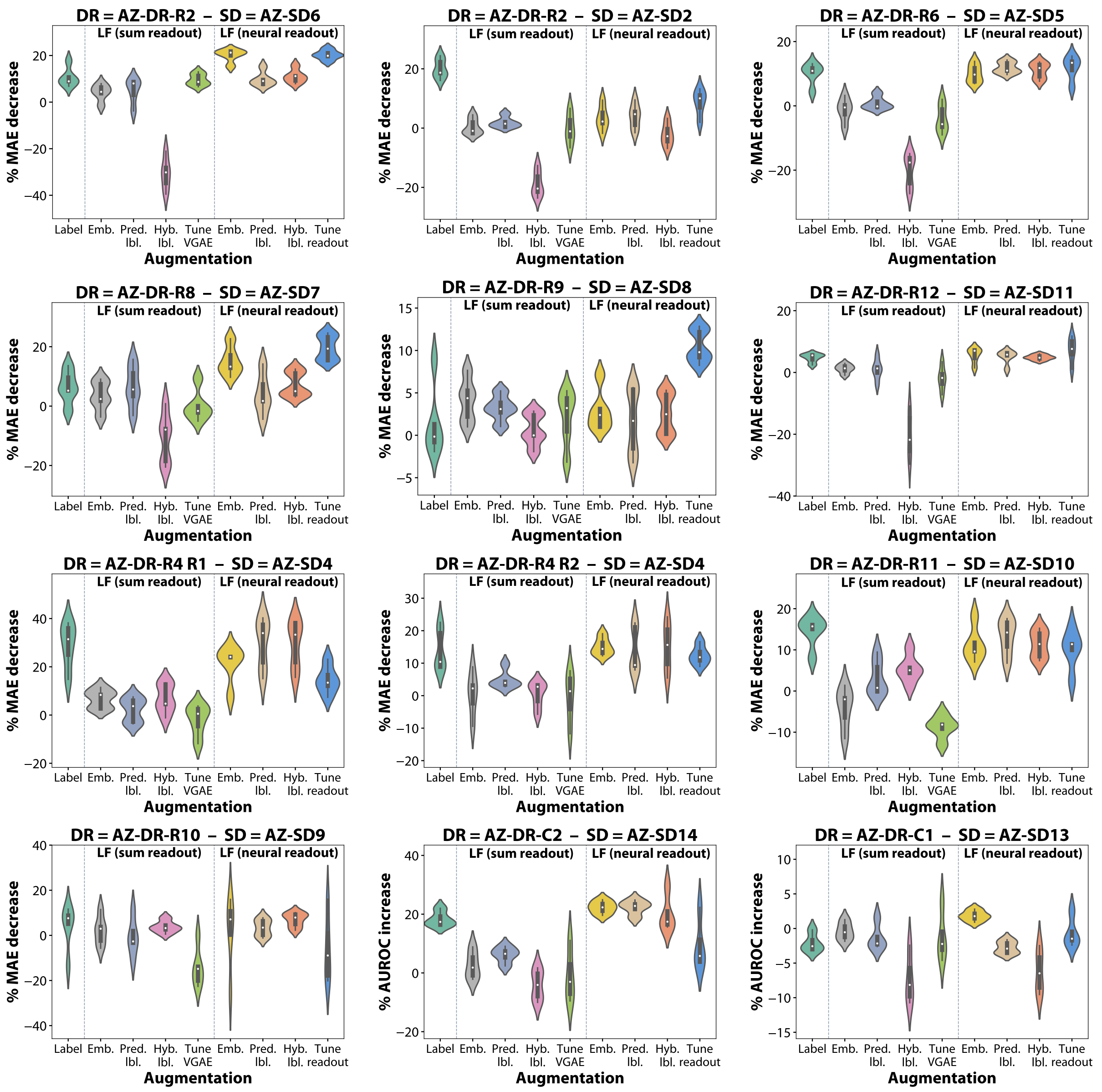

Supplementary Figure 2. Systematic evaluation of AstraZeneca datasets, consisting of high fidelity GNN (VGAE) models with augmentations based on sum and neural readout-based low-fidelity ('LF') models, including fine-tuning (see *Methods*). The results are reported on test sets. The multi-fidelity drug discovery datasets are named based on the high-fidelity (DR, dose response) and low-fidelity (SD, single dose) datasets. The abbreviations are: AZ, AstraZeneca; VGAE, variational graph autoencoder; MAE, mean absolute error; AUROC, area under the receiver operating characteristic. Source data are provided as a Source Data file.

Supplementary Notes 7 Systematic evaluation of transductive low-fidelity augmentations for drug discovery – AZ RF

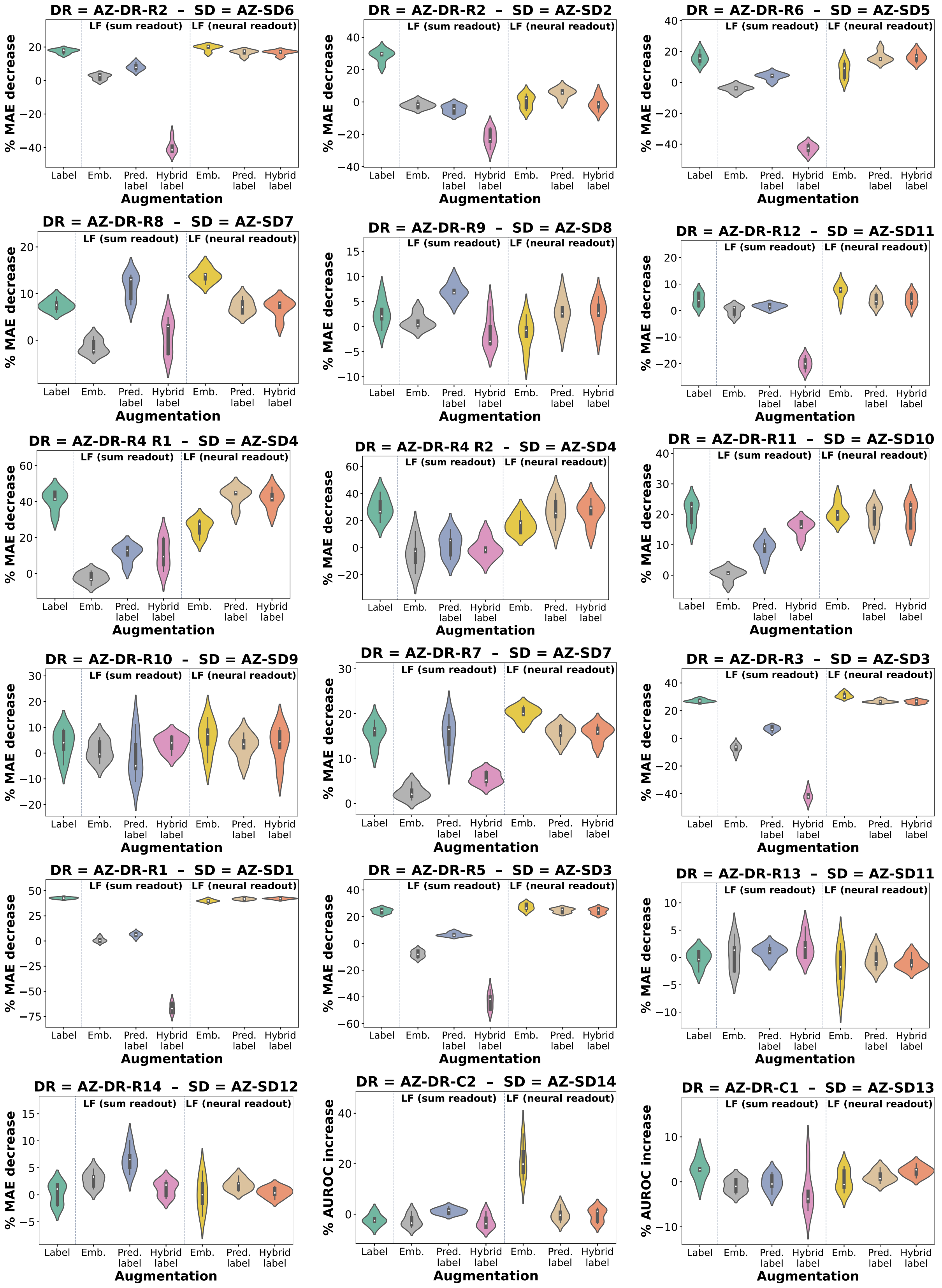

Supplementary Figure 3. Systematic evaluation of AstraZeneca datasets, consisting of high fidelity random forest (RF) models with augmentations based on sum and neural readout-based low-fidelity ('LF') models (see *Methods*). The results are reported on test sets. The multi-fidelity drug discovery datasets are named based on the high-fidelity (DR, dose response) and low-fidelity (SD, single dose) datasets. The abbreviations are: AZ, AstraZeneca; MAE, mean absolute error. Source data are provided as a Source Data file.

Supplementary Notes 8 Systematic evaluation of transductive low-fidelity augmentations for drug discovery – AZ SVM

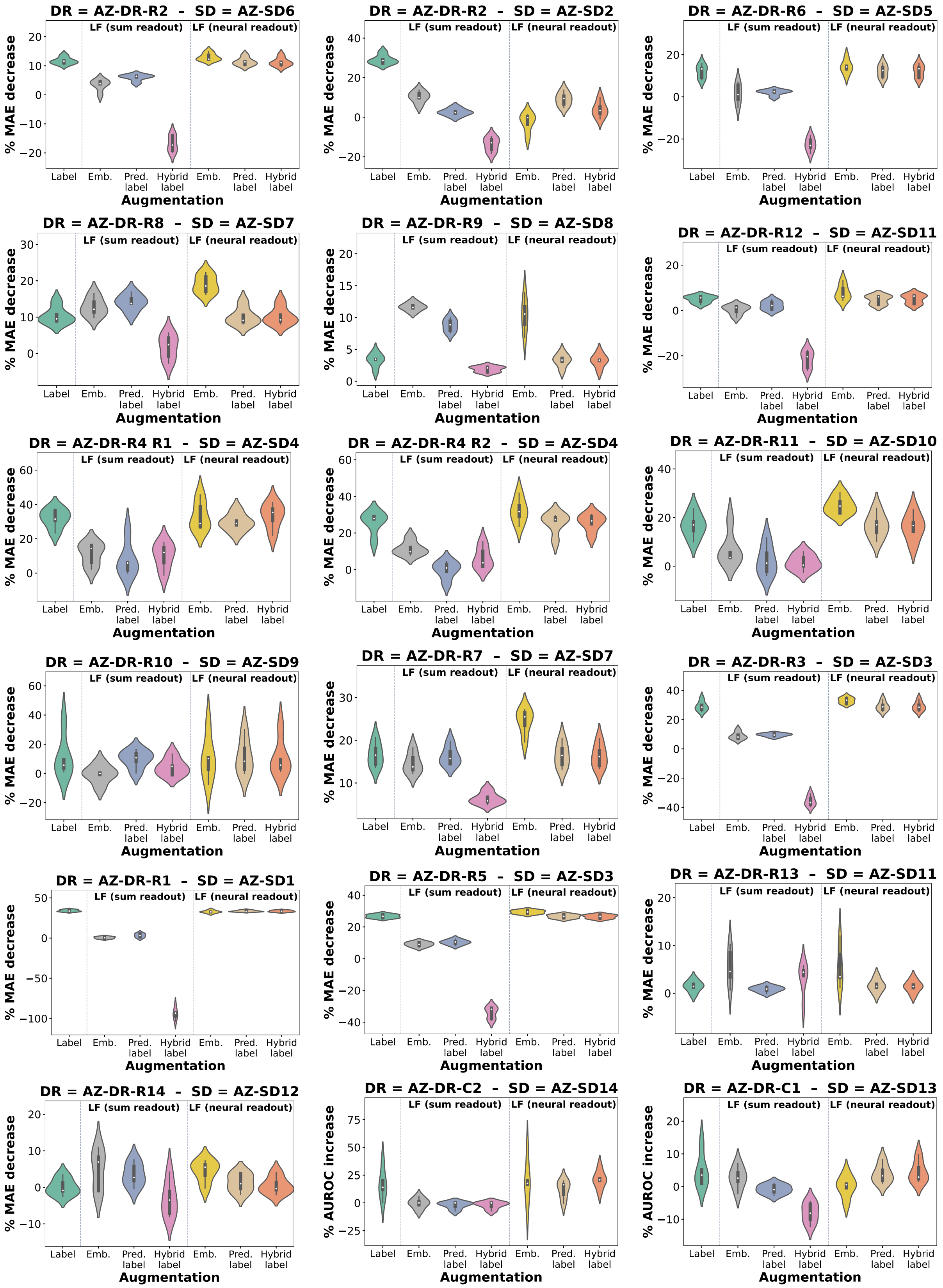

Supplementary Figure 4. Systematic evaluation of AstraZeneca datasets, consisting of high fidelity support vector machine (SVM) models with augmentations based on sum and neural readout-based low-fidelity ('LF') models (see *Methods*). The results are reported on test sets. The multi-fidelity drug discovery datasets are named based on the high-fidelity (DR, dose response) and low-fidelity (SD, single dose) datasets. The abbreviations are: AZ, AstraZeneca; MAE, mean absolute error. Source data are provided as a Source Data file.

Supplementary Notes 9 Systematic evaluation of transductive low-fidelity augmentations for quantum mechanics – GNNs

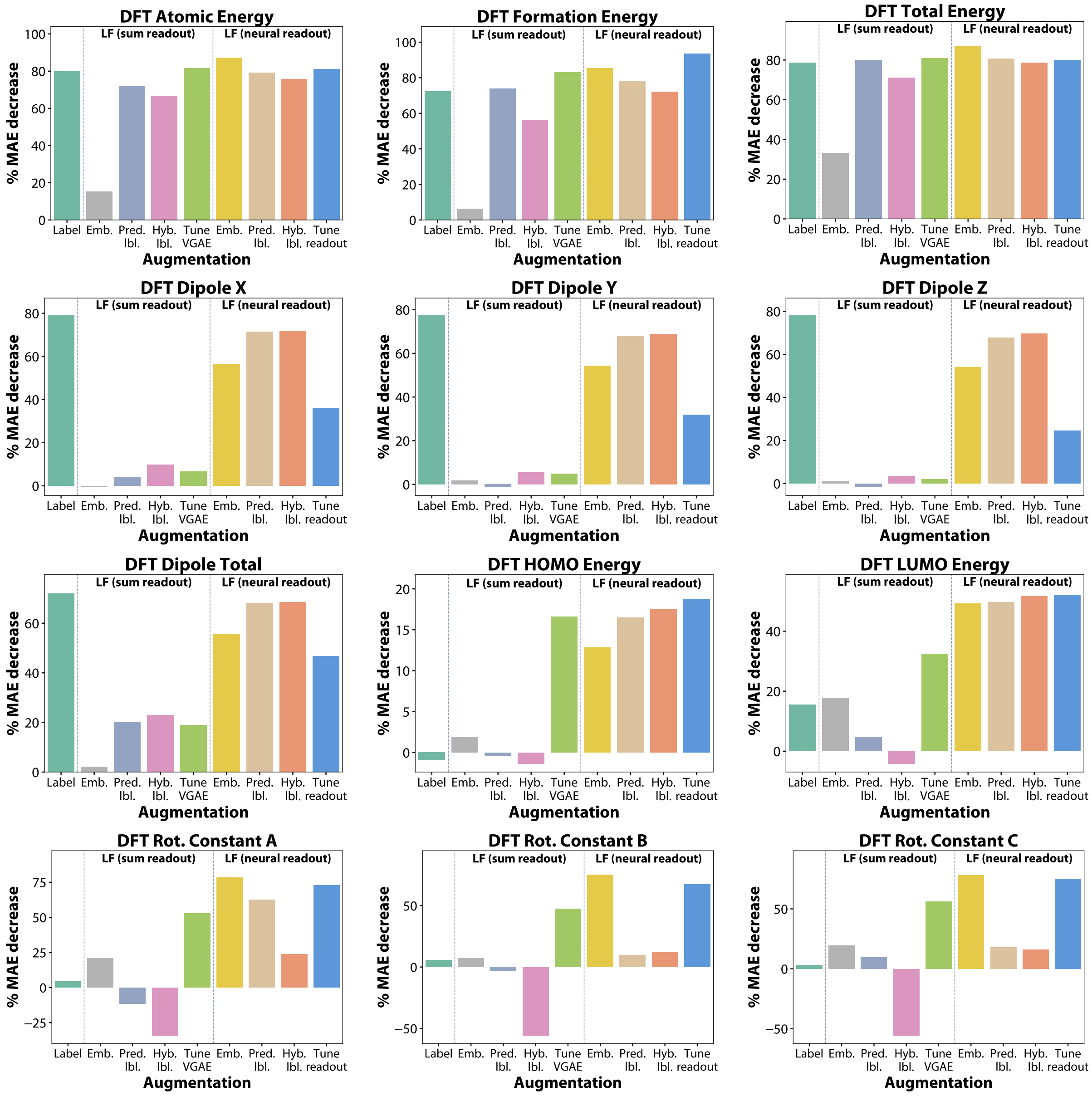

Supplementary Figure 5. Systematic evaluation of QMugs properties, consisting of high fidelity GNN (VGAE) models with augmentations based on sum and neural readout-based low-fidelity ('LF') models, including fine-tuning (see *Methods*). The results are reported on test sets. The abbreviations are: VGAE, variational graph autoencoder; DFT, density functional theory; HOMO, highest occupied molecular orbital; LUMO, lowest unoccupied molecular orbital; MAE, mean absolute error. The shortened term 'Rot.' stands for 'Rotation'. Source data are provided as a Source Data file.

Supplementary Notes 10 Systematic evaluation of transductive low-fidelity augmentations for drug discovery – PubChem GNNs

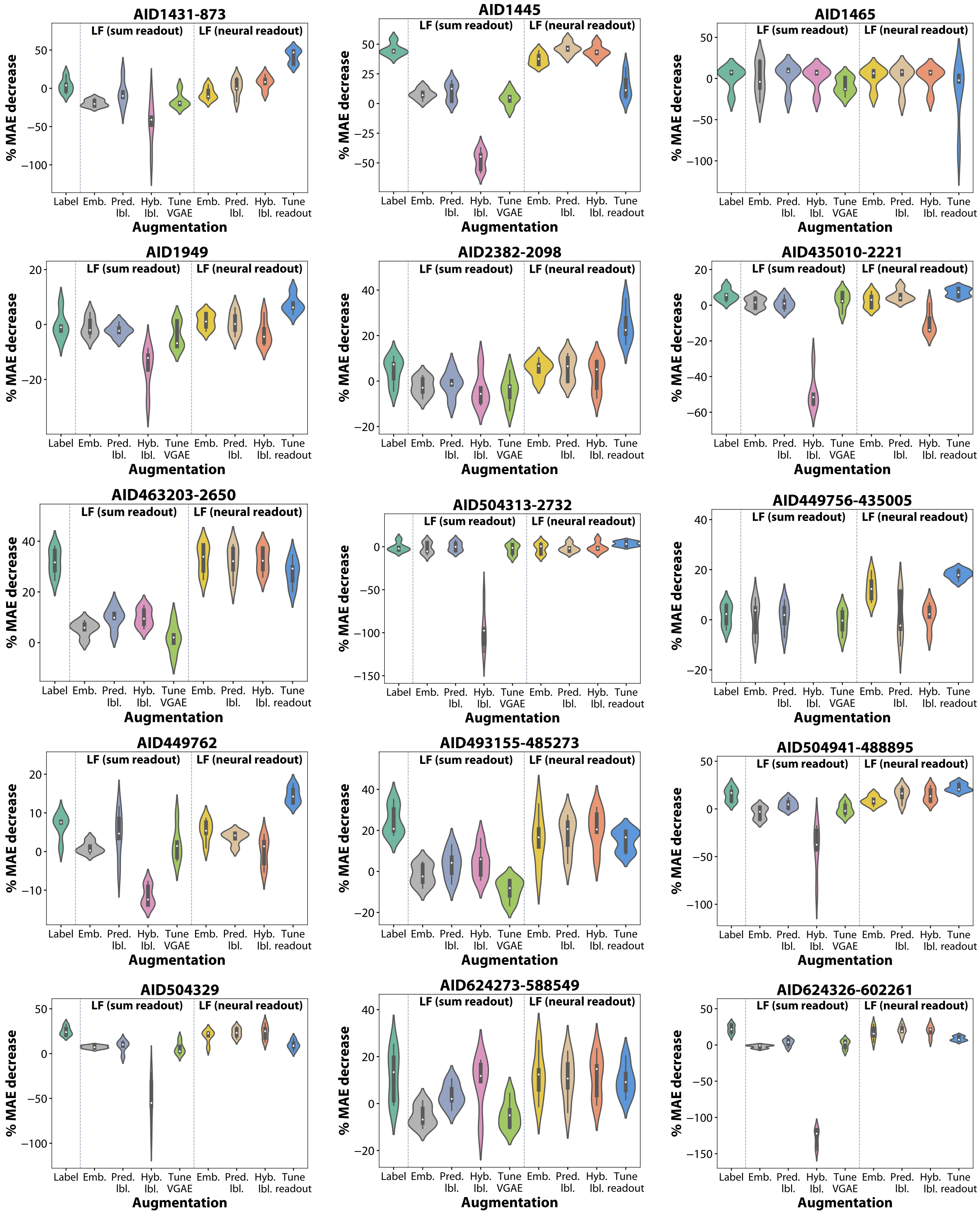

Supplementary Figure 6. Systematic evaluation of PubChem datasets, consisting of high fidelity GNN (VGAE) models with augmentations based on sum and neural readout-based low-fidelity ('LF') models, including fine-tuning (see *Methods*). The results are reported on test sets. The multi-fidelity drug discovery datasets are named based on the high-fidelity (DR, dose response) and low-fidelity (SD, single dose) datasets. The abbreviations are: AID, assay identifier; VGAE, variational graph autoencoder; MAE, mean absolute error. Source data are provided as a Source Data file.

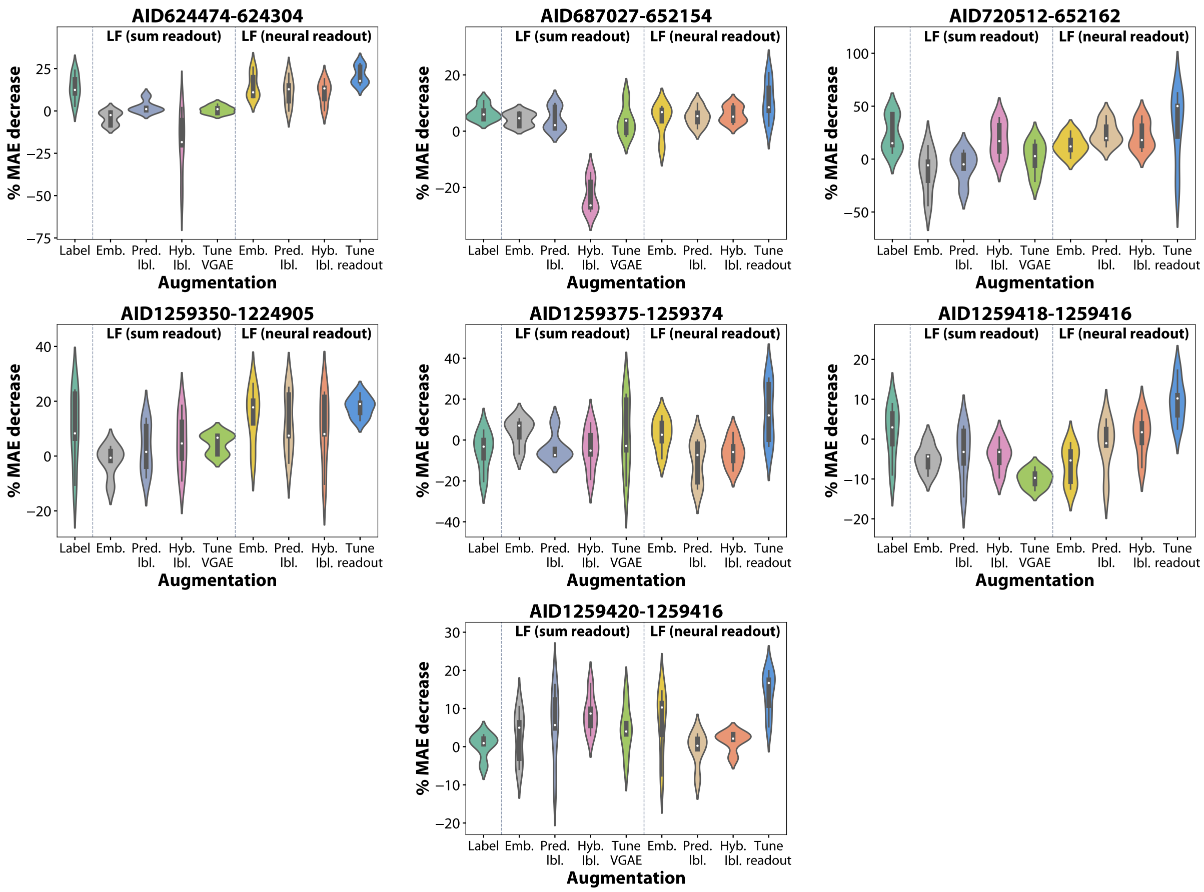

Supplementary Figure 7. Continued from Supplementary Figure 6. The multi-fidelity drug discovery datasets are named based on the high-fidelity (DR, dose response) and low-fidelity (SD, single dose) datasets. The abbreviations are: AID, assay identifier; VGAE, variational graph autoencoder; MAE, mean absolute error. Source data are provided as a Source Data file.

Supplementary Notes 11 Systematic evaluation of transductive low-fidelity augmentations for drug discovery – PubChem RF

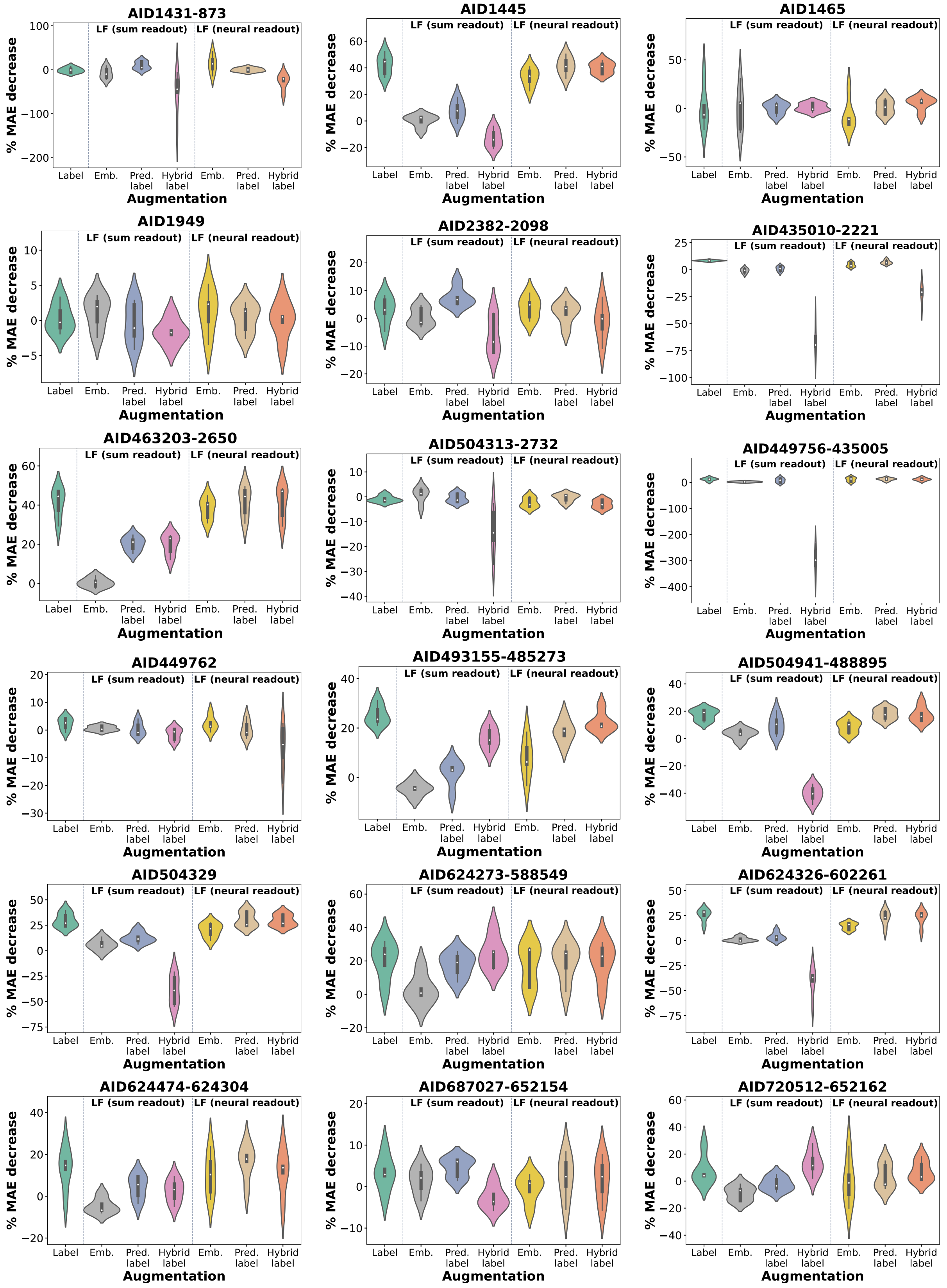

Supplementary Figure 8. Systematic evaluation of PubChem datasets, consisting of high fidelity random forest (RF) models with augmentations based on sum and neural readout-based low-fidelity ('LF') models (see *Methods*). The results are reported on test sets. The multi-fidelity drug discovery datasets are named based on the high-fidelity (DR, dose response) and low-fidelity (SD, single dose) datasets. The abbreviations are: AID, assay identifier; VGAE, variational graph autoencoder; MAE, mean absolute error. Source data are provided as a Source Data file.

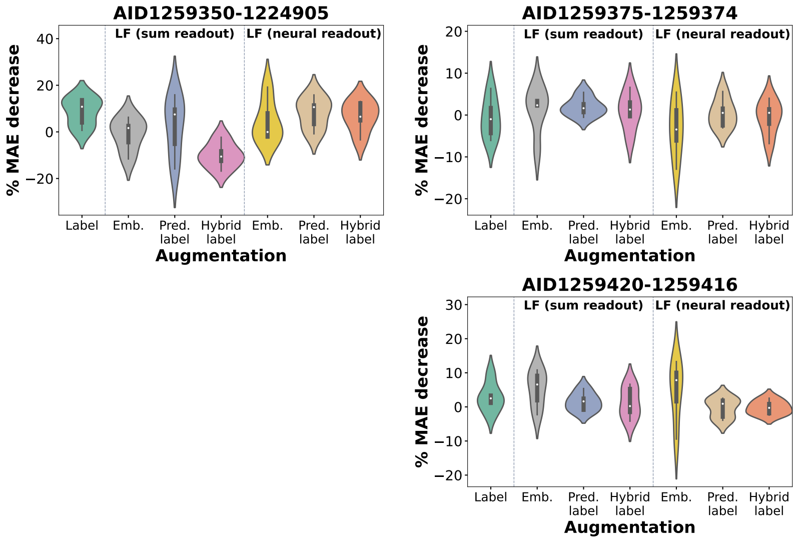

Supplementary Figure 9. Continued from Supplementary Figure 8. The multi-fidelity drug discovery datasets are named based on the high-fidelity (DR, dose response) and low-fidelity (SD, single dose) datasets. The abbreviations are: AID, assay identifier; VGAE, variational graph autoencoder; MAE, mean absolute error. Source data are provided as a Source Data file.

Supplementary Notes 12 Systematic evaluation of transductive low-fidelity augmentations for drug discovery – PubChem SVM

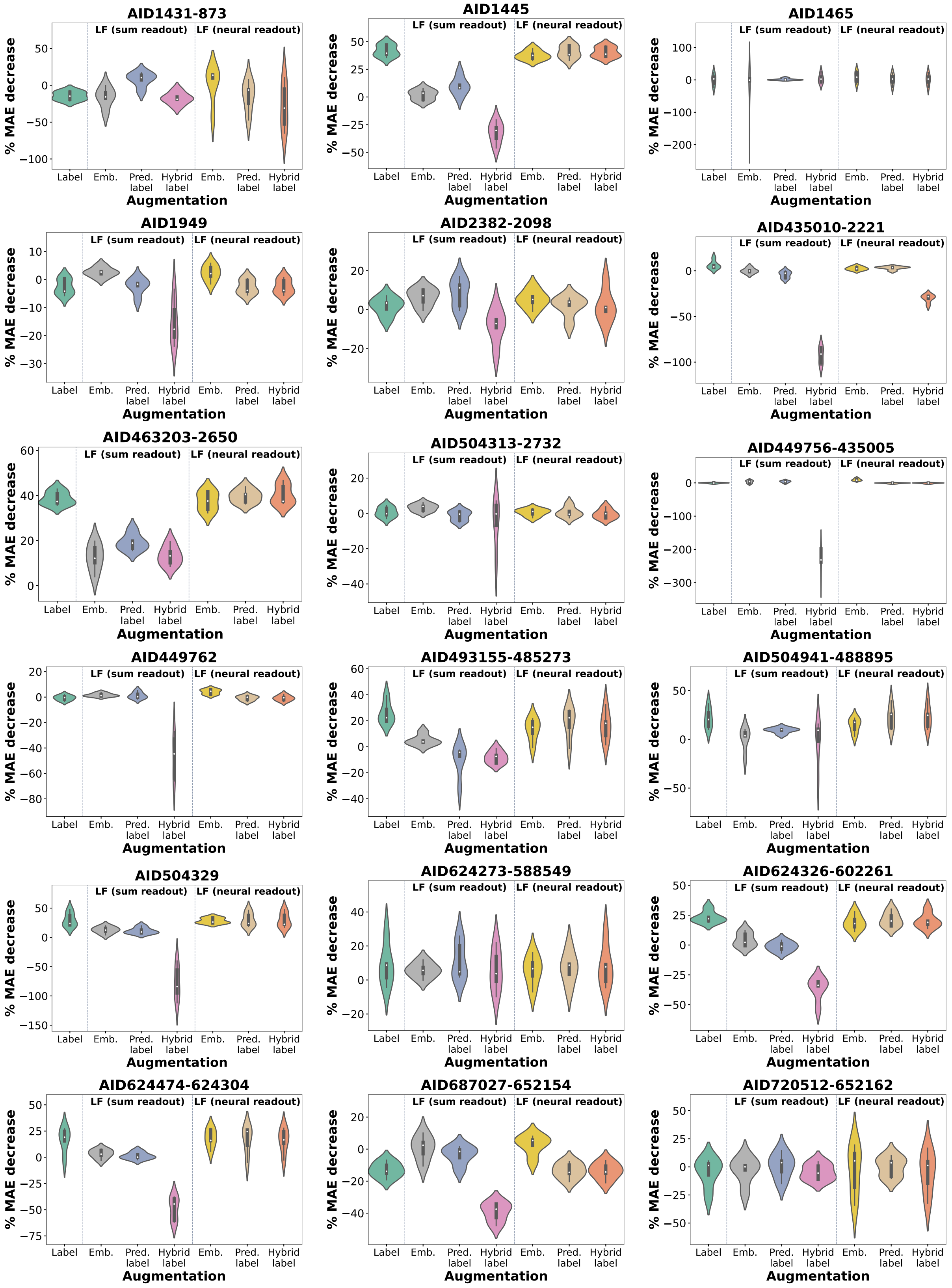

Supplementary Figure 10. Systematic evaluation of PubChem datasets, consisting of high fidelity support vector machine (SVM) models with augmentations based on sum and neural readout-based low-fidelity ('LF') models (see *Methods*). The results are reported on test sets. The multi-fidelity drug discovery datasets are named based on the high-fidelity (DR, dose response) and low-fidelity (SD, single dose) datasets. The abbreviations are: AID, assay identifier; VGAE, variational graph autoencoder; MAE, mean absolute error. Source data are provided as a Source Data file.

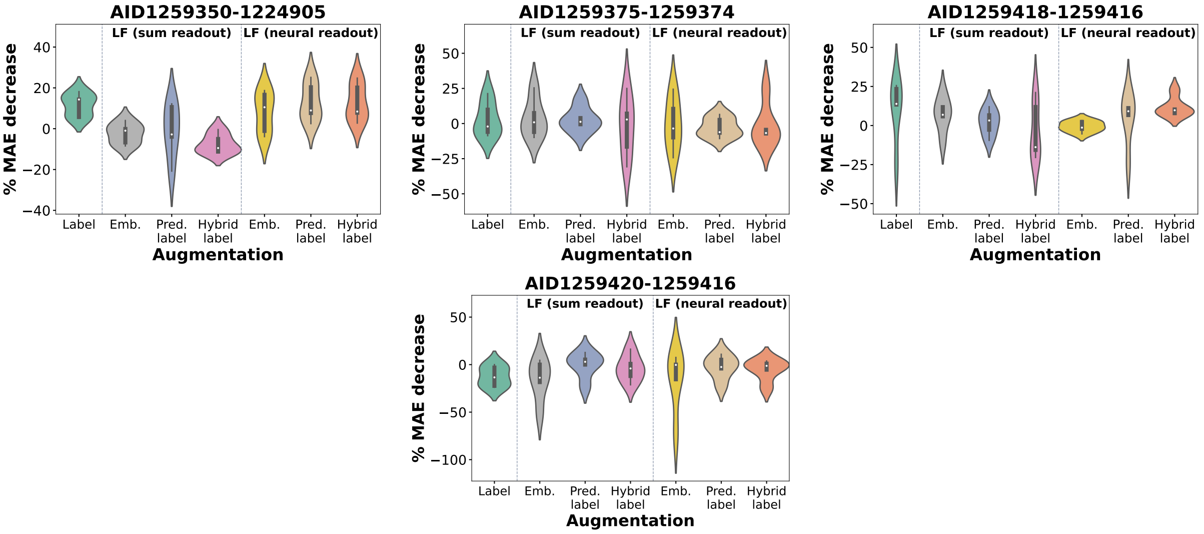

Supplementary Figure 11. Continued from Supplementary Figure 10. The multi-fidelity drug discovery datasets are named based on the high-fidelity (DR, dose response) and low-fidelity (SD, single dose) datasets. The abbreviations are: AID, assay identifier; VGAE, variational graph autoencoder; MAE, mean absolute error. Source data are provided as a Source Data file.

Supplementary Notes 13 Systematic evaluation of inductive low-fidelity augmentations

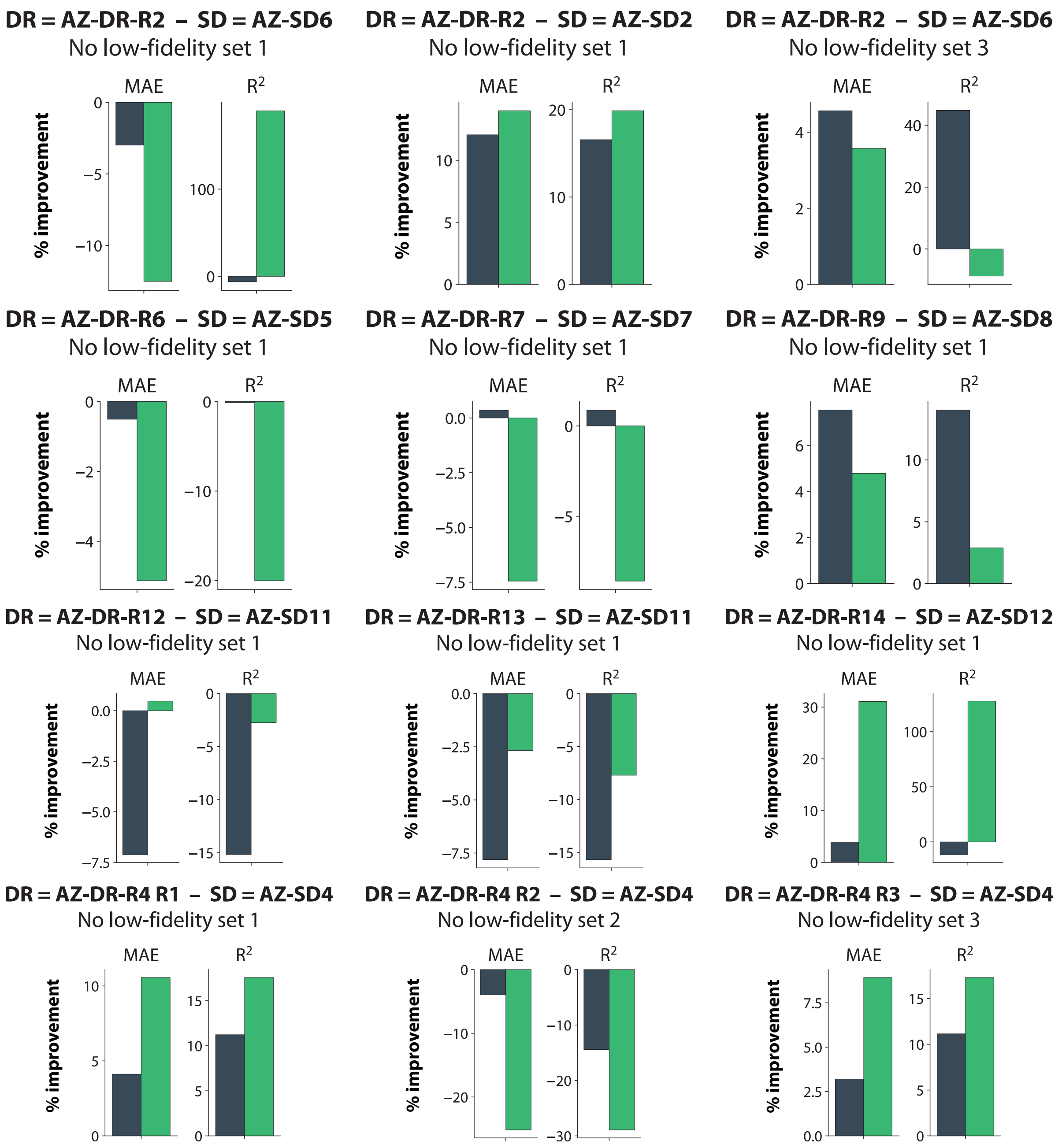

Supplementary Figure 12. Systematic evaluation of high-fidelity models using sum and neural embeddings in an inductive setting. The previous train, validation, and test splits from Figure 4 are used for training and testing is performed on compounds that were measured in subsequent high-throughput screening (HTS) stages. The multi-fidelity drug discovery datasets are named based on the high-fidelity (DR, dose response) and low-fidelity (SD, single dose) datasets. The abbreviations are: AZ, AstraZeneca; MAE, mean absolute error; R^2, coefficient of determination. An additional 'R#' in the multi-fidelity dataset name, such as in the last row, indicates that multiple confirmatory rounds ('R') have been performed in the context of the same project. Source data are provided as a Source Data file.

Supplementary Notes 14 All QMugs training subsets with embeddings augmentations

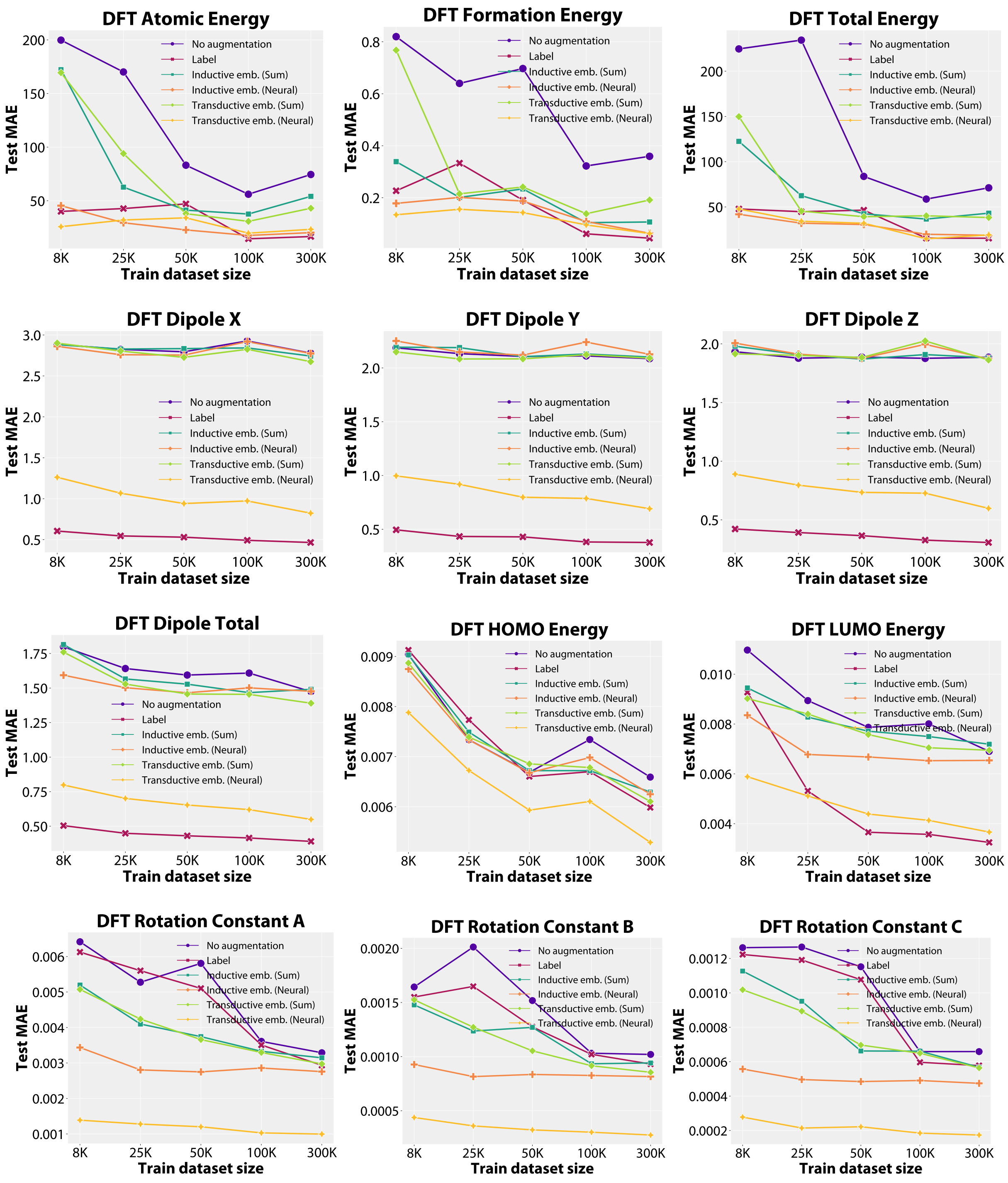

Supplementary Figure 13. Test metrics (MAE) for high-fidelity models with fixed validation and test sets but varying train set sizes and different augmentation strategies based on embeddings. The abbreviations are: DFT, density functional theory; HOMO, highest occupied molecular orbital; LUMO, lowest unoccupied molecular orbital; MAE, mean absolute error. The shortened term 'emb.' stands for 'embeddings'. Source data are provided as a Source Data file.

Supplementary Notes 15 Summary of datasets where the low-fidelity labels are the optimal strategy

Supplementary Table 4. Summary of the 4 PubChem and 2 AstraZeneca multi-fidelity datasets where the low-fidelity labels are the highest ranking transfer learning strategy. The first two numerical columns correspond to the % MAE decrease observed for the high-fidelity models leveraging low-fidelity labels, and for the high-fidelity models with the best adaptive readout ('A.R.') strategy for that particular dataset, respectively. The linear correlation (Pearson's r) between the low-fidelity ('SD') and high-fidelity ('DR') is also listed. The last column corresponds to the decrease in MAE observed between the adaptive readouts and the dataset mean predictor for the low-fidelity training task of Figures 3A and 3B. Bold is used for the table headers.

**Dataset**	**+ LF label (% MAE decrease)**	**+ best A.R. strategy (% MAE decrease)**	**SD/DR $\|r\|$**	**% MAE decrease for LF models**
AID602261-624326	21.94	20.08	0.68	759.97
AID485273-493155	24.70	20.93	0.58	188.84
AID588549-624273	11.75	11.62	0.70	473.44
AID504329	25.04	23.45	0.79	550.49
AZ-DR-R2+AZ-SD-2	20.00	8.65	0.72	130.67
AZ-DR-R11+AZ-SD-10	14.51	13.20	0.30	321.38

From Supplementary Table 4 it is clear that in almost all instances where the labels offer better performance than other strategies, the benefit is only marginal, with the reduction in MAE percentages being very close. Furthermore, it is possible to deduce that two factors contribute to this situation. Firstly, the low-fidelity/high-fidelity (SD/DR) correlation is generally very high (around 0.7 or higher) compared to most multi-fidelity drug discovery datasets, such that the low-fidelity labels carry highly relevant information. A second factor is the performance of the low-fidelity model that is used to generate embeddings or predictions, or to fine-tune the adaptive readout (Figures 3A and 3B). AZ-SD-2 is such a case where the decrease in MAE over the dataset mean predictor is very low (less than two times) compared to the rest of the datasets and to the average (360.32 ± 212.00 for AstraZeneca datasets). A similar case is observed for the public dataset AID485273, where the improvement over the dataset mean is similarly low, especially when compared to the average (464.77 ± 201.84 for PubChem datasets).

For QMugs, the labels are preferable for 4 quantum properties (Table 1). The 4 instances correspond to the 4 dipole properties: 'Dipole X', 'Dipole Y', 'Dipole Z', and 'Dipole Total'. The dipole moment is a property that relies heavily on the geometry of the molecules, and as such message passing algorithms operating on the 2D graph of the molecule are likely to have difficulties. This is a well-known problem in the literature [13, 14]. It is still worth noting that low-fidelity models using adaptive readouts are the only ones which provide performance close to the labels in the transductive case, although in the inductive case all tested methods fail to meaningfully extrapolate to unseen molecules (Supplementary Figure 13).

Supplementary References

1. Weininger, D. SMILES, a chemical language and information system. 1. Introduction to methodology and encoding rules. *Journal of Chemical Information and Computer Sciences* **28,** 31–36. eprint: https://doi.org/10.1021/ci00057a005. https://doi.org/10.1021/ci00057a005 (1988).

2. Buterez, D., Janet, J. P., Kiddle, S. J. & Liò, P. MF-PCBA: Multifidelity High-Throughput Screening Benchmarks for Drug Discovery and Machine Learning. *Journal of Chemical Information and Modeling* **63.** PMID: 37058588, 2667–2678. eprint: https://doi.org/10.1021/acs.jcim.2c01569. https://doi.org/10.1021/acs.jcim.2c01569 (2023).

3. Buterez, D., Janet, J. P., Kiddle, S. J., Oglic, D. & Liò, P. *Transfer learning with graph neural networks for improved molecular property prediction in the multi-fidelity setting* version v0.1. Repository name: multi-fidelity-gnns-for-drug-discovery-and-quantum-mechanics. Dec. 2023. https://doi.org/10.5281/zenodo.10423965.

4. Paszke, A. *et al.* in *Advances in Neural Information Processing Systems 32* 8024–8035 (Curran Associates, Inc., 2019). http://papers.neurips.cc/paper/9015-pytorch-an-imperative-style-high-performance-deep-learning-library.pdf.

5. Fey, M. & Lenssen, J. E. *Fast Graph Representation Learning with PyTorch Geometric* in *ICLR Workshop on Representation Learning on Graphs and Manifolds* (2019).
6. Falcon, W. & The PyTorch Lightning team. *PyTorch Lightning* version 1.4. Mar. 2019. https://github.com/Lightning-AI/lightning.
7. Yang, K. *et al.* Analyzing Learned Molecular Representations for Property Prediction. *Journal of Chemical Information and Modeling* **59.** PMID: 31361484, 3370–3388. eprint: https://doi.org/10.1021/acs.jcim.9b00237. https://doi.org/10.1021/acs.jcim.9b00237 (2019).
8. Stokes, J. M. *et al.* A Deep Learning Approach to Antibiotic Discovery. *Cell* **180,** 688–702.e13. ISSN: 0092-8674. https://www.sciencedirect.com/science/article/pii/S0092867420301021 (2020).
9. Heid, E. *et al.* Chemprop: A Machine Learning Package for Chemical Property Prediction. *ChemRxiv* (2023).
10. Kipf, T. N. & Welling, M. *Semi-Supervised Classification with Graph Convolutional Networks* in *International Conference on Learning Representations* (2017). https://openreview.net/forum?id=SJU4ayYgl.
11. Xu, K., Hu, W., Leskovec, J. & Jegelka, S. *How Powerful are Graph Neural Networks?* in *International Conference on Learning Representations* (2019).
12. Corso, G., Cavalleri, L., Beaini, D., Liò, P. & Velickovic, P. *Principal Neighbourhood Aggregation for Graph Nets* in *Proceedings of the 34th International Conference on Neural Information Processing Systems* (Curran Associates Inc., Vancouver, BC, Canada, 2020). ISBN: 9781713829546.
13. Schütt, K., Unke, O. & Gastegger, M. *Equivariant message passing for the prediction of tensorial properties and molecular spectra* in *Proceedings of the 38th International Conference on Machine Learning* (eds Meila, M. & Zhang, T.) **139** (PMLR, July 2021), 9377–9388. https://proceedings.mlr.press/v139/schutt21a.html.
14. Park, Y. J. *Edge Direction-invariant Graph Neural Networks for Molecular Dipole Moments Prediction* 2022. arXiv: 2206.12867 [cs.LG].
