# Supplementary material for: Transfer learning with graph neural networks for improved molecular property prediction in the multi-fidelity setting
Source: Nat Commun. 2024 Feb 26;15:1517. doi: 10.1038/s41467-024-45566-8 (PMC11258334; doi:10.1038/s41467-024-45566-8)
Supplement: Supplementary file 3 — Reporting Summary [file 41467_2024_45566_MOESM3_ESM.pdf]

nature portfolio

Corresponding author(s): David Buterez
Last updated by author(s): Jan 21, 2024

# Reporting Summary

Nature Portfolio wishes to improve the reproducibility of the work that we publish. This form provides structure for consistency and transparency in reporting. For further information on Nature Portfolio policies, see our Editorial Policies and the Editorial Policy Checklist.

## Statistics

For all statistical analyses, confirm that the following items are present in the figure legend, table legend, main text, or Methods section.

| n/a | Confirmed | |
|---|---|---|
| ☒ | ☐ | The exact sample size (*n*) for each experimental group/condition, given as a discrete number and unit of measurement |
| ☒ | ☐ | A statement on whether measurements were taken from distinct samples or whether the same sample was measured repeatedly |
| ☒ | ☐ | The statistical test(s) used AND whether they are one- or two-sided<br>*Only common tests should be described solely by name; describe more complex techniques in the Methods section.* |
| ☒ | ☐ | A description of all covariates tested |
| ☒ | ☐ | A description of any assumptions or corrections, such as tests of normality and adjustment for multiple comparisons |
| ☒ | ☐ | A full description of the statistical parameters including central tendency (e.g. means) or other basic estimates (e.g. regression coefficient) AND variation (e.g. standard deviation) or associated estimates of uncertainty (e.g. confidence intervals) |
| ☒ | ☐ | For null hypothesis testing, the test statistic (e.g. *F*, *t*, *r*) with confidence intervals, effect sizes, degrees of freedom and *P* value noted<br>*Give P values as exact values whenever suitable.* |
| ☒ | ☐ | For Bayesian analysis, information on the choice of priors and Markov chain Monte Carlo settings |
| ☒ | ☐ | For hierarchical and complex designs, identification of the appropriate level for tests and full reporting of outcomes |
| ☒ | ☐ | Estimates of effect sizes (e.g. Cohen's *d*, Pearson's *r*), indicating how they were calculated |

*Our web collection on statistics for biologists contains articles on many of the points above.*

## Software and code

Policy information about availability of computer code

| | |
|---|---|
| Data collection | This is described in detail in the last section of our code repository's home page: https://github.com/davidbuterez/multi-fidelity-gnns-for-drug-discovery-and-quantum-mechanics |
| Data analysis | This is described in detail in the last section of our code repository's home page: https://github.com/davidbuterez/multi-fidelity-gnns-for-drug-discovery-and-quantum-mechanics |

For manuscripts utilizing custom algorithms or software that are central to the research but not yet described in published literature, software must be made available to editors and reviewers. We strongly encourage code deposition in a community repository (e.g. GitHub). See the Nature Portfolio guidelines for submitting code & software for further information.

## Data

Policy information about availability of data

All manuscripts must include a data availability statement. This statement should provide the following information, where applicable:

- Accession codes, unique identifiers, or web links for publicly available datasets
- A description of any restrictions on data availability
- For clinical datasets or third party data, please ensure that the statement adheres to our policy

The MF-PCBA, QMugs, and QM7b datasets are publicly available and accessible by following the instructions presented in their respective papers. We provide additional instructions relevant to our workflow in our code repository. The proprietary AstraZeneca HTS data collection is not publicly available. For the purposes of this work, the proprietary data are pre-processed and used within our computational workflow following identical steps to MF-PCBA. Source data are provided with

this paper (for all Figures and Supplementary Figures, with the exception of the UMAP plots of Figure 3C). Due to the large size, the UMAP data is instead hosted on the GitHub repository listed below.

## Research involving human participants, their data, or biological material

Policy information about studies with human participants or human data. See also policy information about sex, gender (identity/presentation), and sexual orientation and race, ethnicity and racism.

| | |
|---|---|
| Reporting on sex and gender | N/A |
| Reporting on race, ethnicity, or other socially relevant groupings | N/A |
| Population characteristics | N/A |
| Recruitment | N/A |
| Ethics oversight | N/A |

Note that full information on the approval of the study protocol must also be provided in the manuscript.

# Field-specific reporting

Please select the one below that is the best fit for your research. If you are not sure, read the appropriate sections before making your selection.

[x] Life sciences [ ] Behavioural & social sciences [ ] Ecological, evolutionary & environmental sciences

For a reference copy of the document with all sections, see nature.com/documents/nr-reporting-summary-flat.pdf

# Life sciences study design

All studies must disclose on these points even when the disclosure is negative.

| | |
|---|---|
| Sample size | Not relevant |
| Data exclusions | What would apply for our paper is the filtering of some molecules in the dataset. This is described in detail in the manuscript, as the filtering procedure for the datasets is involved. |
| Replication | Multiple (5) runs for each datasets/model were done, and the results were reported as mean and standard deviations. |
| Randomization | We used a standard machine learning train/validation/test splitting strategy in the relevant parts of the paper. |
| Blinding | Not relevant |

# Reporting for specific materials, systems and methods

We require information from authors about some types of materials, experimental systems and methods used in many studies. Here, indicate whether each material, system or method listed is relevant to your study. If you are not sure if a list item applies to your research, read the appropriate section before selecting a response.

## Materials & experimental systems

| n/a | Involved in the study | |
|---|---|---|
| x | | Antibodies |
| x | | Eukaryotic cell lines |
| x | | Palaeontology and archaeology |
| x | | Animals and other organisms |
| x | | Clinical data |
| x | | Dual use research of concern |
| x | | Plants |

## Methods

| n/a | Involved in the study | |
|---|---|---|
| x | | ChIP-seq |
| x | | Flow cytometry |
| x | | MRI-based neuroimaging |

## Plants

| | |
|---|---|
| Seed stocks | Not relevant |
| Novel plant genotypes | Not relevant |
| Authentication | Not relevant |
